# Data Mining of a Remote Behavioral Tracking System for Type 2 Diabetes Patients: A Prospective Cohort Study

**DOI:** 10.2196/diabetes.4506

**Published:** 2016-04-06

**Authors:** Noah Wayne, Nick Cercone, Jiye Li, Ariel Zohar, Joel Katz, Patrick Brown, Paul Ritvo

**Affiliations:** 1 Health Behaviour Change Lab School of Kinesiology & Health Science York University Toronto, ON Canada; 2 Data Mining Lab Lassonde School of Engineering York University Toronto, ON Canada; 3 Pain Mechanisms Lab Department of Psychology York University Toronto, ON Canada; 4 Division of Biostatistics Dalla Lana School of Public Health University of Toronto Toronto, ON Canada; 5 Analytics and Informatics Prevention and Cancer Control Cancer Care Ontario Toronto, ON Canada

**Keywords:** diabetes mellitus, type 2, health coaching, mhealth, telehealth, data mining

## Abstract

**Background:**

Complications from type 2 diabetes mellitus can be prevented when patients perform health behaviors such as vigorous exercise and glucose-regulated diet. The use of smartphones for tracking such behaviors has demonstrated success in type 2 diabetes management while generating repositories of analyzable digital data, which, when better understood, may help improve care. Data mining methods were used in this study to better understand self-monitoring patterns using smartphone tracking software.

**Objective:**

Associations were evaluated between the smartphone monitoring of health behaviors and HbA1c reductions in a patient subsample with type 2 diabetes who demonstrated clinically significant benefits after participation in a randomized controlled trial.

**Methods:**

A priori association-rule algorithms, implemented in the C language, were applied to app-discretized use data involving three primary health behavior trackers (exercise, diet, and glucose monitoring) from 29 participants who achieved clinically significant HbA1c reductions. Use was evaluated in relation to improved HbA1c outcomes.

**Results:**

Analyses indicated that nearly a third (9/29, 31%) of participants used a single tracker, half (14/29, 48%) used two primary trackers, and the remainder (6/29, 21%) of the participants used three primary trackers. Decreases in HbA1c were observed across all groups (0.97-1.95%), but clinically significant reductions were more likely with use of one or two trackers rather than use of three trackers (OR 0.18, *P*=.04).

**Conclusions:**

Data mining techniques can reveal relevant coherent behavior patterns useful in guiding future intervention structure. It appears that focusing on using one or two trackers, in a symbolic function, was more effective (in this sample) than regular use of all three trackers.

## Introduction

Diabetes is a cluster of metabolic conditions characterized by dysglycemia from defects in insulin secretion and/or unhealthy behaviors that cause debilitating complications and death [1,2]. In 2013, an estimated 8.3% of the global population lived with diabetes and more than US $612 billion was spent on treatment [3]. A type 2 diabetes (T2DM) diagnosis, affecting 90-95% of people with diabetes, largely results from genetic predisposition, excess body weight, physical inactivity, and poor diet [1]. Additional studies demonstrate that lower socioeconomic status (SES) populations are at greater risk for developing diabetes [4,5] and often demonstrate poorer disease management, resulting in more frequent (and more expensive) complications and hospitalizations [5].

There is consensus among researchers and clinical professionals that glucose self-monitoring, exercise, and diet-related health behaviors are important in effective T2DM management and well-regulated serum glucose levels [6]. Monitoring health behaviors (ie, exercise, healthy diet, glucose monitoring) may be important in improving glucose control [6]. Mobile tracking technologies can help patients adopt and sustain self-management behaviors and can help health professionals provide better monitoring and support. However, there are challenges in determining the optimal design of self-tracking tools and their optimal use with respect to frequency and duration.

Data mining (DM) refers to analytic approaches useful in detecting coherent patterns in large and complex datasets [7]. The applications of DM methods in analyses of diabetes-related health behaviors are continually being improved, especially in the selection of analytic frameworks that capture key data with sufficient explanatory power [8].

As utilization of electronic health records increases in health care, DM becomes more relevant [9] to chronic disease prevention and management [8]. A recent study utilized descriptive DM algorithms to analyze a dataset with 450 attributes to identify a “short list” of the behavioral correlates of depressive disorder [10]. This study exemplifies use of DM in datasets lacking the uniformity needed for more conventional analyses [11]. DM can help integrate variables of multiple types (eg, diet, exercise, blood pressure, SES, income, geographic location) in better understanding factors affecting diabetes incidence, prevalence, and management [7].

In another diabetes study, DM algorithms were used to construct a model that predicted short-term changes in blood glucose [12] exemplifying how DM can help identify risk factors for hypoglycemia [13]. Several studies found that information collected on meals, insulin therapy, and physical activity improved the prediction of blood glucose levels [12-14]. Applying DM analyses to self-monitoring data could help identify key indicators in the prevention of life-threatening hypoglycemic events [15]. DM applied to primary medical data could point to useful methods for initiating and maintaining effective T2DM treatment, leading to better resource allocation and treatment personalization [16,17].

Health coaching is a promising clinical role that stimulates and supports health behavior change in patients with varying SES, health problems and diagnosed chronic diseases. When connected with 24 hour/day/7 day/week mobile phone-based counseling, health coaching is associated with benefits for individuals affected by uncontrolled T2DM [18-20] and chronic obstructive pulmonary disease [17].

### Objective

Our primary objective was to evaluate associations between the mobile phone monitoring of health behaviors, within a randomized controlled trial (RCT), and clinically significant reductions in glycated hemoglobin (HbA1c).

## Methods

The RCT protocol was reviewed and approved by the York University research ethics board (Certificate #2012-033), and all patients provided written, informed consent to participate.

**Figure 1:**
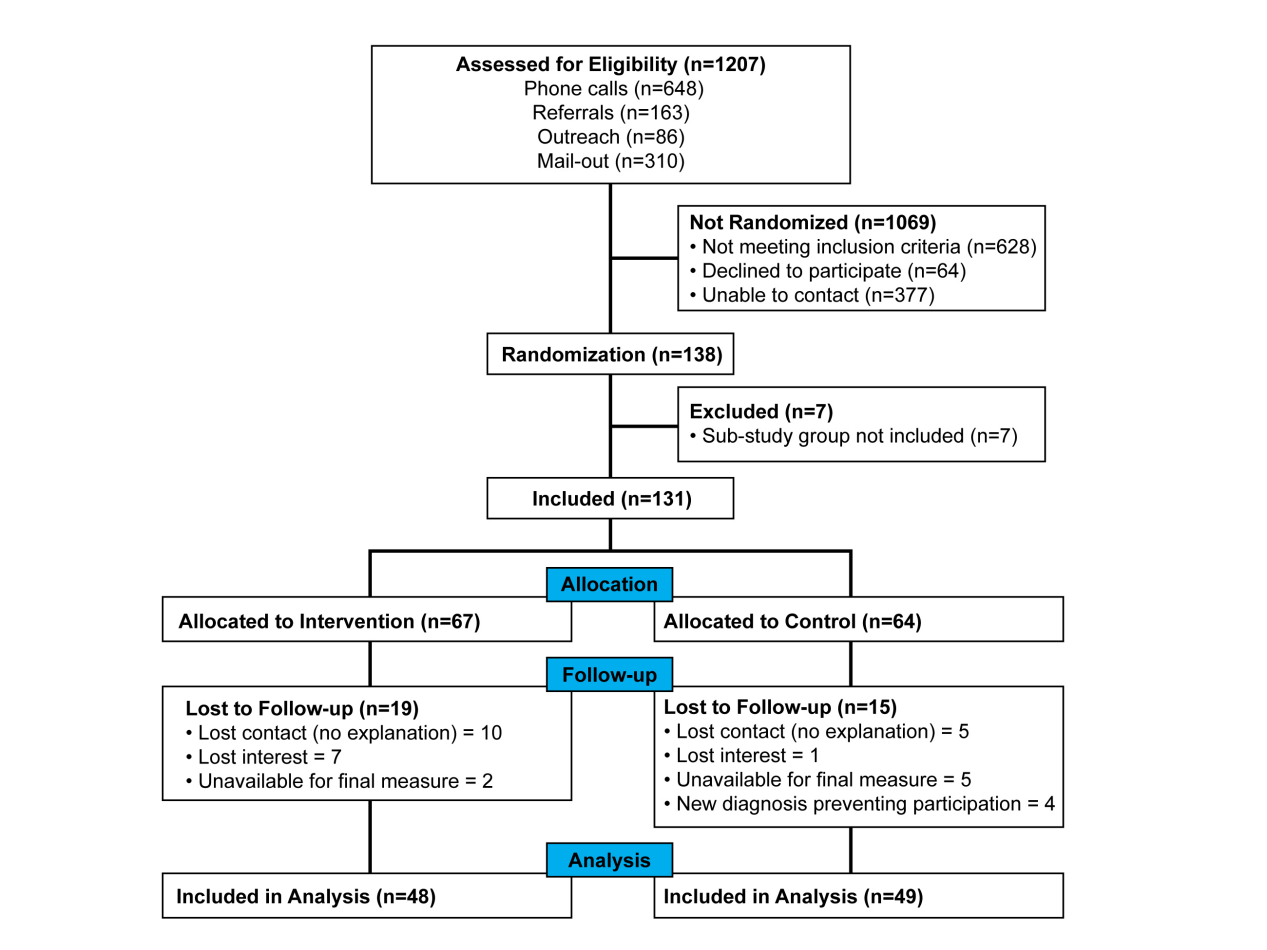
Flowchart of enrollment.

The RCT assessed T2DM patients (N=97) assisted by personal health coaches trained in behavior-change theories, practices, and counseling methods (see Figure 1). At baseline, all patients had poor glucose regulation as indicated by glycated hemoglobin (HbA1c) ≥7.3%. In the mobile phone–assisted intervention arm, participants (n=48) were provided a smartphone (Samsung Galaxy Ace 2) with data service and pre-installed health tracking software (NexJ Systems Inc., Connected Wellness Platform [CWP]) enabling the detailed monitoring of two behaviors (exercise and diet) and a risk-related outcome (blood glucose) throughout a 24-week intervention. Health coaches helped patients use the mobile phone software in ways that best fit their daily routines.

CWP use data were extracted from NexJ Systems servers upon trial completion and compiled into .csv files stored on password-protected portable drives. Study participant IDs were matched with software user IDs, as data were anonymized and prepared for analyses.

### Data Analysis

#### Association Rule Algorithms

To discover useful relationships between self-tracker use and HbA1c outcomes, we employed association-rule algorithms software to find coherent relationships in transactions represented by sets of items, termed “frequent item sets.” For example, when customer A buys bread and cheese, and customer B buys bread, cheese and burgers, bread and cheese appear frequently on both shopping lists. Therefore, bread and cheese are associated and they qualify as a frequent item set [bread, cheese]. Support is a term reflecting the measurement of association frequency, as defined by the percentage of observations to which the item sets belong. In our study, support was defined as the number of times an attribute value (such as *a1c 6 month diff=1.1*) or a set of attribute values (such as *glucose_count=200* and *food_count=150*) appear in participant data, divided by the total number of participants, expressed in a percentage (ie, multiplied by 100).

We used association rule algorithms to identify all common attributes in participants. The support threshold was fixed at a minimum of 5%, such that item sets were generated that occurred in at least 5% of the sample. We used the a priori association rule algorithm implemented by C language for the Unix/Linux environment.

#### Attribute Selection

Among the 97 T2DM study completers, the present analysis considers patients in the experimental group (n=48). As our objective was to evaluate associations between the use frequency of different trackers in the CWP in relation to HbA1c outcomes, we selected the change in HbA1c over 24 weeks (*a1c6month_diff*) and 4 software uses as the attributes for the association rule algorithm. These attributes were use frequency of the blood glucose tracker (*glucose_count*), use frequency of the food tracker (*food_count*), use frequency of the exercise tracker (*exercise_count*), and the total use frequency of all three trackers (*generic_count*).

#### Discretization

We proceeded to discretize the attributes to implement the association rule algorithm. For the four tracker attributes (*glucose_count*, *food_count*, *exercise_count*, and *generic_count*), data were discretized into categories relevant to typical use during a standard week, with numerical criteria selected to reflect significant adoption levels by patients (see Table 1). Discretization was compiled differently per tracker to align with expected and actual adoption rates per tracked behavior (or outcome). For example, glucose management in T2DM includes self-monitoring of serum-glucose via finger prick, recommended several times daily for poorly managed patients, and less often if there is better gluco-regulation. Use of the blood glucose tracker was discretized based on frequencies of 1-4.9 uses per week, 5-7.9 uses per week, 8-13.9 uses per week, and 14-21.9 uses per week. For food tracking, a similar discretization pattern was used. Since use frequency fluctuated, discretization was calculated as up to once per week, 1-3.9 uses per week, 4-6.9 uses per week, 7-13.9 uses per week (1 to <2/day), 14-20.9 uses per week (2 to <3/day), and 21+ per week (3+/day). Since the exercise tracker was the least frequently used, discretization rules were adjusted to once every other week, 0.5-0.9 uses per week, 1-1.9 uses per week, 2-2.9 uses per week, 3-3.9 uses per week, and 4<6 uses per week. The total use of all trackers was discretized to once per week, 1-3.9 times per week, 4-6.9 times per week (0.5-1/day), 7-13.9 times per week (1-2/day), 14-17.9 times per week (2-4/day), 18-34.9 times per week (4-5/day), and 35-50 times per week (5-7/day).

**Table 1:** Discretization of tracker use frequency ^a-d^.

Attribute	Group	Range	Meaning
Glucose count	1	0-0	Zero
	2	5-24	Up to once per week
	3	24-120	1-4.9 times/week
	4	120-192	5-7.9 times/week
	5	192-336	8-13.9 times/week
	6	336-520	14-21.9 times/week
Exercise count	1	0-0	Zero
	2	0-5	Minimal (>0-4)
	3	5-12	Once every other week
	4	12-24	0.5-0.9 time/week
	5	24-48	1-1.9 times/week
	6	48-72	2-2.9 times/week
	7	72-96	3-3.9 times/week
	8	96-150	4-6 times/week
Food count	1	0-0	Zero
	2	1-5	>0-5 over 6 months
	3	5-24	Up to 1 time/week
	4	24-96	1-3.9 times/week
	5	96-168	4-6.9 times/week (1/day)
	6	168-336	7-13.9 times/week (1-1.9/day)
	7	336-504	14-20.9 times/week (2-2.9/day)
	8	504-720	21+ times/week (3+/day)
Generic count	1	0-0	Zero
	2	0-24	1 time/week
	3	24-96	1-3.9 times/week
	4	96-168	4-6.9 times/week (0.5-1/day)
	5	168-336	7-13.9 times/week (1-2/day)
	6	336-672	14-17.9 times/ week (2-4/day)
	7	672-840	18-34.9 times/ week (4-5/day)
	8	840-1200	35-50 times/week (5-7/day)

^a^Attribute=type of tracker used.

^b^Group=discretization group.

^c^Range=frequencies of use for allocation to group.

^d^Meaning=frequency of use in terms of use per week/day.

#### Preprocessing for HbA1c Attribute

For the *a1c6month_diff* attribute, conceived of as the most important attribute in investigating the desired associations, we preserved its original value to an optimal degree. Therefore, we did not discretize this attribute but created an extension of attributes based on the *a1c6month_diff* attribute. We obtained a set of all possible values for *a1c6month_diff* attribute (a), such as {-1.4, -0.8, -0.5, -0.3, -0.1, 0, 0.1, 0.2, 0.5, a_n_}. For patients with an *a1c6month_diff* value of a_1_ through to a_n_, we created the following new attributes: *a1c6_at_least_-1.4*, *a1c6_at_least_-0.8*, *a1c6_at_least_-0.5*, ..., *a1c6_at_least_n*. Accordingly, the patient with the lowest reduction value was associated with only one attribute value (of this kind) while the patient with the highest reduction was associated with all the attribute values established.

#### Postprocessing of Associations

The above pre-processing approaches produced redundancies in the input data for the association rule algorithm. Therefore, we applied three approaches to post-processing after the associations were generated. First, if a frequent item set contained only *a1c6_at_least_a*_*n*_ attribute values, where a_n_ is a float number (from a_1_ to a_n_), we removed such item sets (for example, *a1c6_at_least_0.4 a1c6_at_least_0.2* and *a1c6_at_least_-0.3 a1c6_at_least_-0.8 a1c6_at_least_-1.4*). The preceding two frequent item sets were thus removed because the extra *a1c6_at_least* values were added by the pre-processing approach were not relevant unless generated together with the other attributes.

Second, for a frequent item set, if *a1c6_at_least_a*_*n*_ the attribute values that appeared multiple times were removed except the *a1c6_at_least_a*_*n*_ where n is the largest value among the “a1c6” values, (for example, *glucose_120 a1c6_at_least_0.5 a1c6_at_least_0.3*)*.*

In this frequent item set, the *a1c6_at_least* attribute occurred twice. However, when a reduction value for “a1c6” of at least 0.5 exists, by default, the reduction value of 0.3 must exist. Therefore, *a1c6_at_least_0.3* was a redundant attribute value and was therefore removed. The above frequent item set becomes the following after post-processing: *glucose_120 a1c6_at_least_0.5*.

Third, after considering the above two situations, redundant item sets existed in the result. For example, *glucose_120 a1c6_at_least_0.7 a1c6_at_least_0.4* and *glucose_120 a1c6_at_least_0.7 a1c6_at_least_0.1*. These two frequent item sets both contained redundant “*a1c6_at_least*” attribute values. After considering the second situation, these two frequent item sets became *glucose_120 a1c6_at_least_0.7* and *glucose_120 a1c6_at_least_0.7*. Since they were identical and their support values were identical, we removed one of them.

Pre- and postprocessing were implemented by Python using the Linux environment. Attribute selection and categorization were used to determine how system use was associated with change in HbA1c per participant. A minimum clinically significant change approach was used to determine what proportion of participants demonstrated 0.5% (5.5 mmol/mol) or greater reductions of HbA1c and used trackers at variable levels of intensity [21]. Therefore, patient data were extracted only with associations containing the attribute-value pair *a1c_0.5*, representing changes in HbA1c values that were 0.5% or above, which was true for 29 participants.

Last, a Fisher’s exact test for count data was applied to determine the statistical relationship between use of all three trackers, use of one or two trackers, and HbA1c reductions. For this test, the 10 intervention participants who used the software but did not have a clinically significant reduction in HbA1c were used as a comparison group. Results were considered significant at the *P*=.05 level.

## Results

### Usage Data

**Figure 2:**
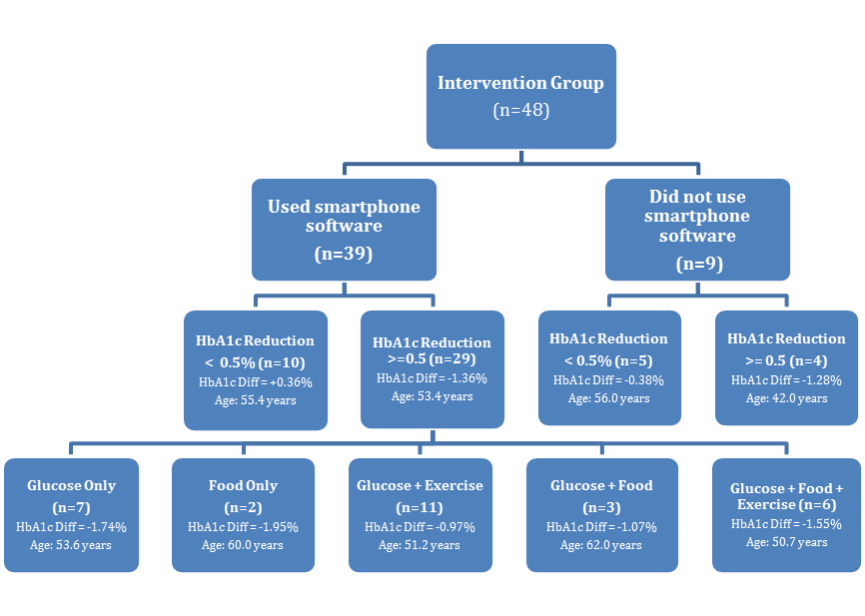
Breakdown of smartphone usage group.

**Figure 3:**
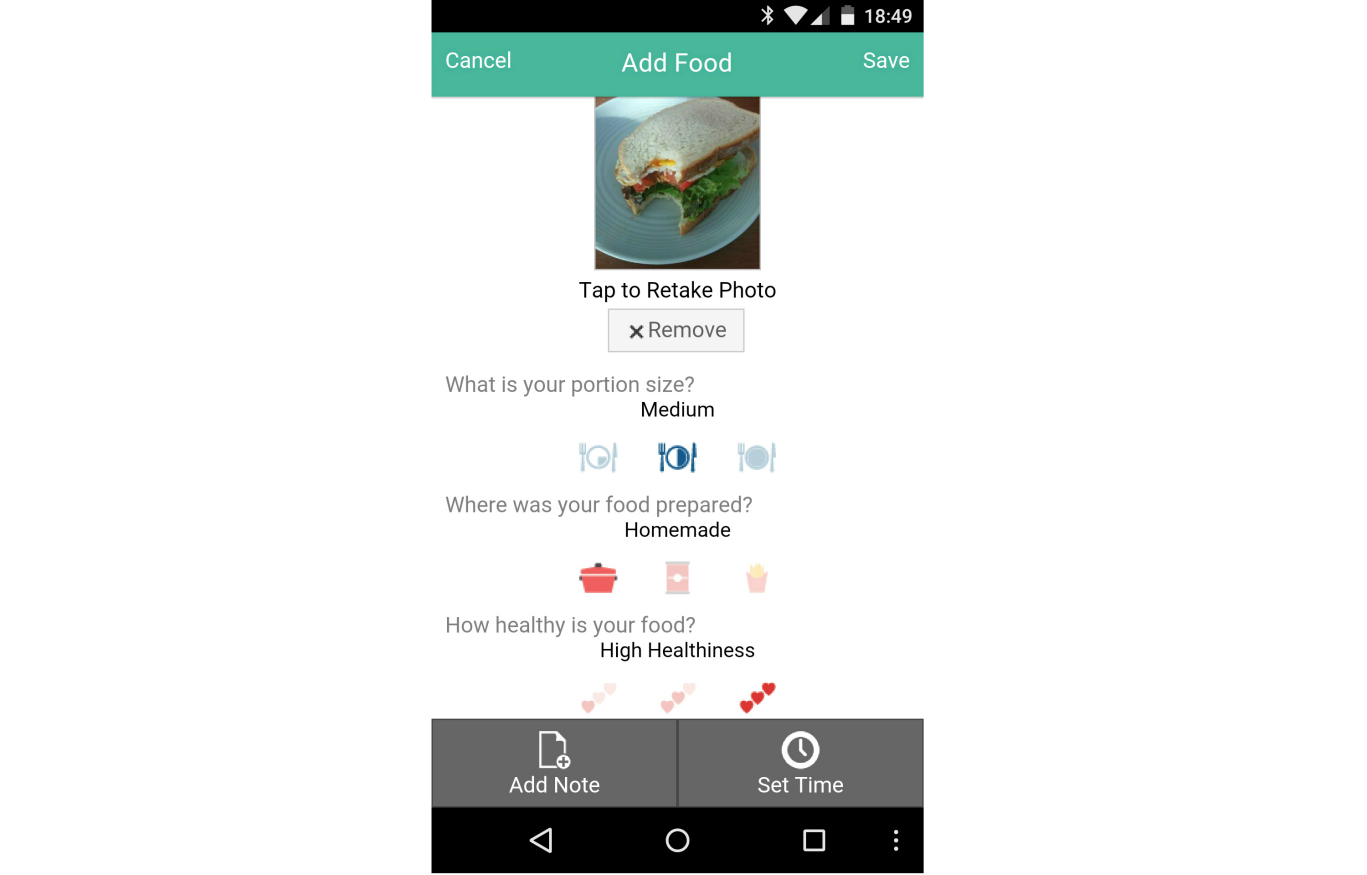
Food tracker.

**Figure 4:**
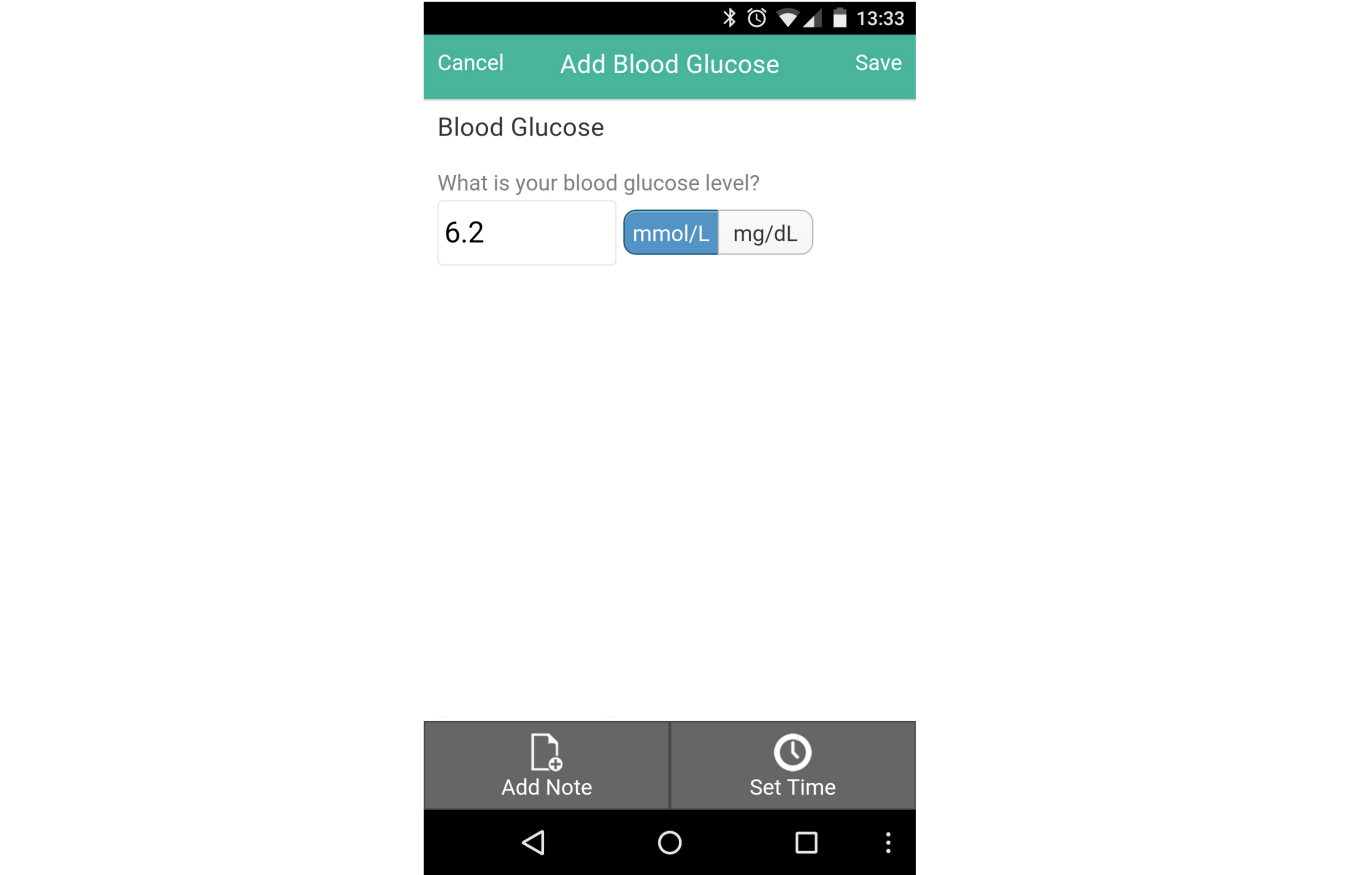
Blood glucose tracker.

**Figure 5:**
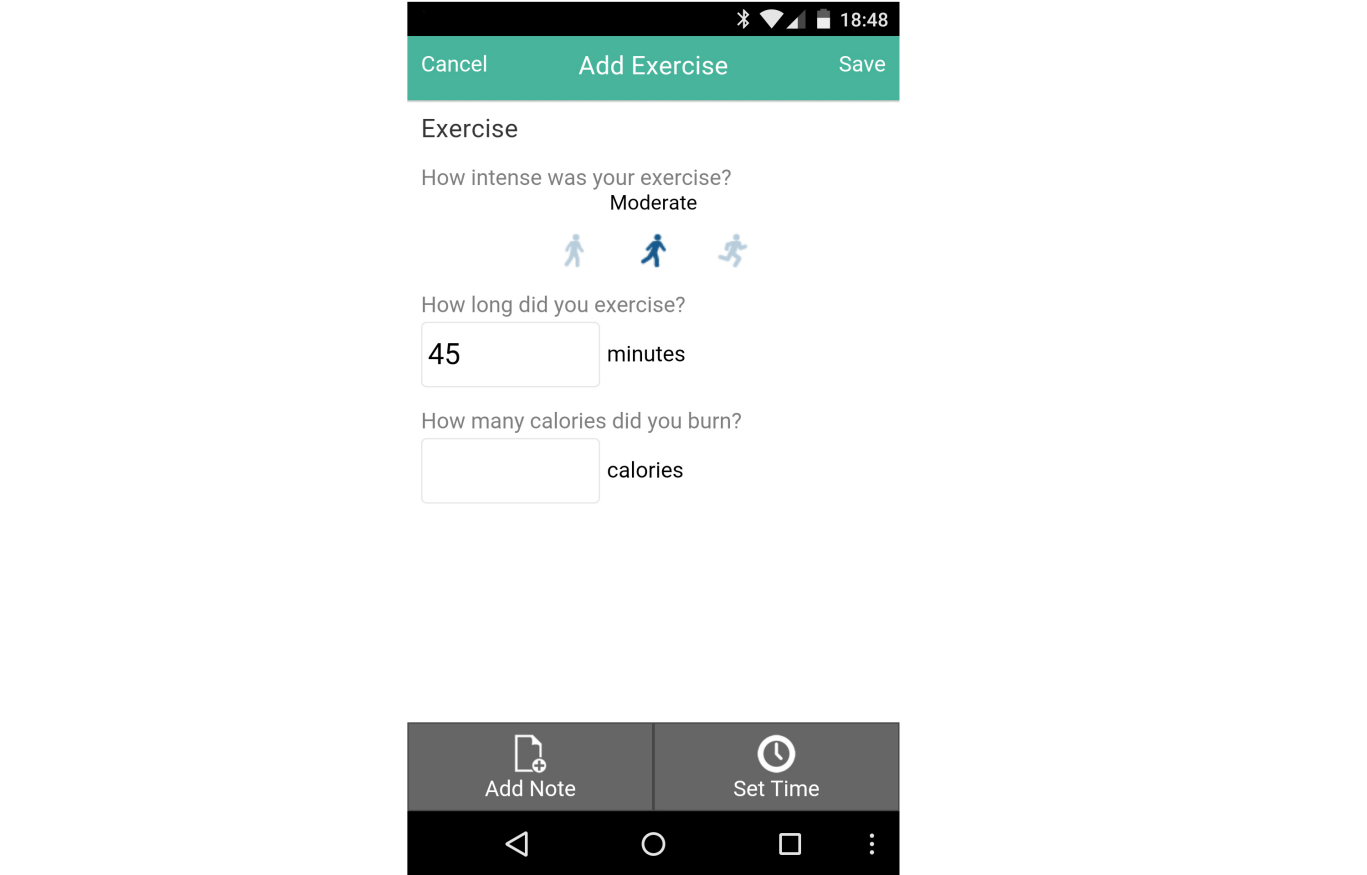
Exercise tracker.

In total, 48 intervention patients completed the 24-week trial. Only 39 patients used the CWP software to track health behaviors. Of this software software-user sample, 29 reduced their HbA1c measure by clinically significant levels at trial conclusion, defined as 0.5% (5.5 mmol/mol) or greater (see Figure 2). Demographic and baseline characteristics of the clinically significant HbA1c reduced-users are provided in Table 2 and compared with the full intervention sample. While multiple health trackers were available in the CWP, most participants used one or a combination of two-to-three specific trackers (ie, between (1) glucose monitoring, (2) exercise tracking, and (3) food tracking) (see Figures 3-5). These trackers reflect the behaviors often considered relevant in diabetes management and therefore the trackers often recommended by health coaches for patients use. Of the 29 clinically significant HbA1c reduced-user group (selected as a subpopulation of interest [SOI]) (HbA1c reduction ≥0.5%), 2 singularly used the food tracker and 7 singularly used the glucose tracker, while no subjects singularly used the exercise tracker. Meanwhile, 11 of these subjects used both glucose and exercise trackers, 3 used both glucose and food trackers, while 0 subjects used both food and exercise trackers. Last, 6 subjects used all three trackers.

**Table 2:** Subject demographics.

		SOI (n=29)	Full intervention sample (n=48)
Age in years, mean (range) SD		53.4 (26-68) 10.7	53.1 (26-74) 10.9
**HbA1c, %** (mmol/mol) SD			
	Baseline	8.88% (73.6) 1.30	8.69% (71.5) 1.32
	6 months	7.52% (58.7) 0.95	7.88% (62.6) 1.17
	Reduction (baseline to 6 months)	1.36% (14.9)	0.81% (8.9)
**Sex, n (%)**			
	Male	11 (33)	17 (35)
	Female	18 (66)	31 (65)
** Education, n (%)**			
	Less than high school	5 (17)	10 (21)
	High school	14 (48)	17 (35)
	College diploma or vocational training	6 (21)	11 (23)
	University degree	4 (14)	8 (17)
	Did not disclose	0 (0)	2 (4)
** Car access, n (%)**			
	Owns a car	10 (34)	19 (40)
	Has access to car	8 (28)	9 (19)
	No access to car	11 (38)	19 (40)
	Not disclosed	0 (0)	1 (2)
** Employment status, n (%)**			
	Unemployed	10 (35)	16 (33)
	Student	1 (3)	3 (6)
	Part-time	0 (0)	1 (2)
	Full-time	8 (28)	13 (27)
	Retired	3 (10)	6 (13)
	Self-employed	5 (17)	6 (13)
	Work in the home (take care of children)	2 (7)	2 (4)
	Not disclosed	0 (0)	1 (2)
** Income in CAD$, n (%)**			
	0-9999	4 (14)	9 (19)
	10,000-25,000	8 (28)	10 (21)
	25,000-50,000	7 (24)	12 (25)
	50,000-75,000	2 (7)	3 (6)
	75,000-up	3 (10)	4 (8)
	Did not disclose	5 (17)	10 (21)

As seen in Table 3, the HbA1c reductions of subjects who used single trackers (food-tracker only or glucose-tracker only) did not significantly differ from each other. More subjects (11/48, 22.9%) used the glucose and exercise tracker in combination than the glucose and food tracker in combination (3/48, 6.3%), but there were no significant differences in HbA1c levels in these subjects. The 6 of 48 subjects (12.5%) who used all three trackers (glucose/food/exercise) achieved a mean HbA1c reduction of 1.55%.

The Fisher’s exact test for count data indicated subjects who used the software (n=39) were more likely to achieve a clinically significant reduction in HbA1c if they used one or two trackers than if they used all three trackers (OR 0.18, *P*=.04) (see Table 4).

**Table 3:** Tracker usage pattern.

Mobile phone tracker	Users, n	Mean reduction of HbA1c, %	SD
Food only	2	1.95	1.20
Glucose only	7	1.74	1.00
Exercise only	0	0	0
Glucose + exercise	11	0.97	0.38
Glucose + food	3	1.07	0.45
Food + exercise	0	0	0
Glucose + food + exercise	6	1.55	0.49

**Table 4:** Fisher’s exact test for count data on three trackers versus less^a^.

	Achieved clinical HbA1c reduction (≥0.5%)	Did not achieve clinical HbA1c reduction (<0.5%)
All 3 trackers	6	6
<3 trackers	23	4

^a^OR 0.18 and *P*=.04.

### Data Mining

The glucose tracker was the most frequently used CWP function. Altogether, 22.9% (11/48) of subjects tracked their blood glucose with the software 1-4.9 times per week *and* had a clinically significant reduction in HbA1c (>0.5%) (see Table 5) while an additional 22.9% with >0.5% reduction in HbA1c demonstrated more frequent glucose tracker use (6/48, 12.5%) and used it 5-7.9 times per week while 10.4% (5/48) used it 8-13.9 times per week.

The food tracking function followed a similar pattern, although with slightly less frequent use. We found 9/48 (18.8%) of participants who achieved at least a 0.5% reduction in HbA1c used the food tracker a minimal amount (one time or less per week), while 7/48 (14.6%) used the system 1-3.9 times per week, and 4/48 (8.3%) used the system 4-6.9 times per week (see Table 6).

**Figure 6:**
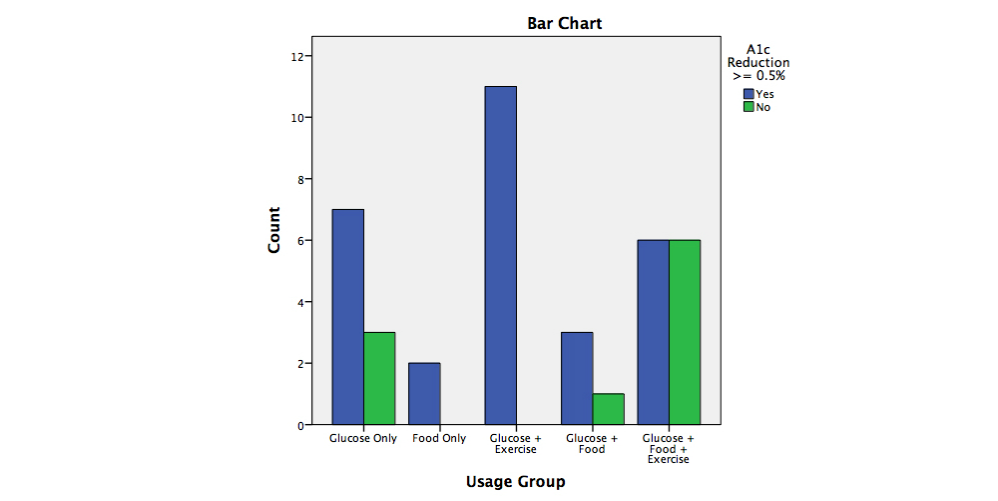
Tracker usage group by clinical HbA1c reduction (ÃƒÂ¢Ã¢â‚¬Â°Ã‚Â¥0.5%).

The least used tracker (and the only tracker that was never singularly used) was the exercise tracker, which was used by 7/48 (14.6%) of the intervention participants between 0.5-0.9 times per week (Tables 7 and 8). When it was paired with glucose tracking, however, every patient who used the exercise tracker had clinically significant reductions in HbA1c (see Figure 6).

**Table 5:** Glucose tracker use.

HbA1c diff.	Glucose 1-4.9 times/week		Glucose 5-7.9 times/week		Glucose 8-13.9 times/week	
	Users, %	n	Users, %	n	Users, %	n
0.1	29.2	14	12.5	6	12.5	6
0.2	27.1	13	12.5	6	12.5	6
0.3	25	12	12.5	6	12.5	6
0.4	22.9	11	12.5	6	12.5	6
0.5	22.9	11	12.5	6	10.4	5
0.6	20.8	10	10.4	5	10.4	5
0.7	18.8	9	10.4	5	10.4 8	5
0.8	16.7	8	10.4	5	10.4	5
0.9	16.7	8	10.4	5	10.4	5
1.1	14.6	7	10.4	5	8.3	4
1.2	10.4	5	8.3	4		
1.3	10.4	5	8.3	4		
1.5	8.3	4	8.3	4		

**Table 6:** Food tracker use.

HbA1c diff.	Food up to 1x/wk		Food 1-3.9x/wk		Food 4-6.9x/wk	
	Users, %	n	Users, %	n	Users, %	n
0.1	20.8	10	16.7	8	8.3	4
0.2	18.8	9	16.7	8	8.3	4
0.3	18.8	9	14.6	7	8.3	4
0.4	18.8	9	14.6	7	8.3	4
0.5	18.8	9	14.6	7	8.3	4
0.6	16.7	8	12.5	6	8.3	4
0.7	16.7	8	10.4	5	8.3	4
0.8	16.7	8	10.4	5	8.3	4
0.9	14.6	7	8.3	4	8.3	4
0.11	14.6	7				
0.12	8.3	4				
0.13	8.3	4				

**Table 7:** Exercise tracker use—Part 1.

HbA1c diff.	Exercise up to 1x/wk		Exercise <5x in 6 months		Exercise 1x every other wk	
	Users, %	n	Users, %	n	Users, %	n
0.1	16.7	8	16.7	8	14.6	7
0.2	16.7	8	14.6	7	14.6	7
0.3	14.6	7	14.6	7	14.6	7
0.4	14.6	7	12.5	6	12.5	6
0.5	14.6	7	12.5	6	12.5	6
0.6	12.5	6	12.5	6	8.3	4
0.7	12.5	6	12.5	6	8.3	4
0.8	12.5	6	12.5	6	8.3	4
0.9	12.5	6	10.4	5	8.3	4
0.11	12.5	6	10.4	5	8.3	4
0.13	8.3	4	10.4	5		
0.15			8.3	4		

**Table 8:** Exercise tracker use—Part 2.

HbA1c diff.	Exercise 0.5-0.9x/wk		Exercise 1-1.9x/wk		Exercise 2-2.9x/wk	
	Users, %	n	Users, %	n	Users, %	n
0.1	14.6	7	8.3	4	8.3	4
0.2	14.6	7	8.3	4	8.3	4
0.3	14.6	7			8.3	4
0.4	14.6	7			8.3	4
0.5	14.6	7				
0.6	14.6	7				
0.7	12.5	6				
0.8	12.5	6				
0.9	10.4	5				
0.11	8.3	4				

## Discussion

### Principal Findings

In the emergent field of mobile phone–assisted health coaching, a recurring question is whether the expense of integrating mHealth technology justifies the benefits. This highlights the need to further investigate for whom (which subpopulations) these technologies are useful and which technologies are most useful for them. In addressing these questions, this study pilots a method of investigating RCT participants, focusing on those who derived clinically significant benefit from participation *and* made significant use of the mobile phone software. The study specifies how much use they engaged in, and the associated benefits, using the clinically significant reduction in HbA1c criteria of 0.5% (5.5 mmol/mol) or greater.

By employing attribute categorizations, tracker-use frequencies were determined per subject in an SOI of significant users who also derived clinically significant glucose reductions. This dataset enabled comparison of subjects who used single trackers versus multiple trackers (two or three trackers). Descriptive results indicated that in subjects achieving a 0.5% HbA1c reduction or better, singular use of the glucose and food monitoring was undertaken, while, in contrast, in the same group, no singular use of the exercise tracker was undertaken. In dual-tracker use, glucose and exercise trackers were employed by more subjects than glucose and food tracking. Interestingly, food and exercise (as dual) trackers were not used by any of the subjects who met criteria for 0.5% HbA1c reduction or greater. Last, all three trackers were used by 12.5% (6/48) of subjects and were associated with a substantive HbA1c mean reduction (1.55% or 16.9 mmol/mol).

The Fisher’s exact test for count data indicated that for subjects who used the software (n=39) and used all three trackers, there was a significantly lower likelihood of achieving a clinically significant reduction in HbA1c than for those who used a lesser number (ie, one or two trackers) (OR 0.18, *P*=.04). This finding indicates that it might be advisable for patients with type 2 diabetes to focus on one or two trackers, especially if one tracker assesses blood glucose levels. From a behavioral perspective, these data could influence health coaching recommendations for health behavior tracking.

### Limitations

Data mining is often used to process large amounts of data. One limitation of the pilot application of data mining in this study was the relatively small user sample size. Nonetheless, the association rule algorithm technique offers a foundation with which to study larger datasets of mHealth tracking technologies as they become available. In terms of diabetes intervention, this was an RCT of typical size (48 intervention participants) and, altogether 10,695 uses of the mobile phone app were analyzed (about 62 uses per month per participant who used the software). Although future DM studies may address larger datasets, this pilot demonstrated application in an RCT dataset within which >10,000 app uses were analyzed (averaging ~1 use per day).

### Conclusion

In summary, this study points to a future when the mobile monitoring of health behaviors will increase and provide digital signals representing engagement in discrete behaviors and daily-weekly-monthly outcomes. Whereas previous associations between counseling and outcomes were difficult to obtain and often based on retrospective self-report, mobile phone monitoring offers ongoing records that precisely reflect status improvements, their stability, and fluctuations (eg, relapsing patterns). Altogether, with the increasing collection of wearable data, we may derive a quantifiable perspective on health changes that instructs the patient and health coach in improving chronic disease management.

## Abbreviations

CWP: Connected Wellness Platform
DM: data mining
HbA1c: glycated hemoglobin
RCT: randomized controlled trial
SOI: subpopulation of interest
T2DM: type 2 diabetes mellitus
